# DepITCM: an audio-visual method for detecting depression

**DOI:** 10.3389/fpsyt.2024.1466507

**Published:** 2025-01-23

**Authors:** Lishan Zhang, Zhenhua Liu, Yumei Wan, Yunli Fan, Diancai Chen, Qingxiang Wang, Kaihong Zhang, Yunshao Zheng

**Affiliations:** ^1^ Key Laboratory of Computing Power Network and Information Security, Ministry of Education, Shandong Computer Science Center, Qilu University of Technology (Shandong Academy of Sciences), Jinan, China; ^2^ Shandong Provincial Key Laboratory of Computer Networks, Shandong Fundamental Research Center for Computer Science, Jinan, China; ^3^ Shandong Mental Health Center, Shandong University, Jinan, China

**Keywords:** depression detection, multimodal, feature extraction, multi-task learning, DepITCM

## Abstract

**Introduction:**

Depression is a prevalent mental disorder, and early screening and treatment are crucial for detecting depression. However, there are still some limitations in the currently proposed deep models based on audio-video data, for example, it is difficult to effectively extract and select useful multimodal information and features from audio-video data, and very few studies have been able to focus on three dimensions of information: time, channel, and space at the same time in depression detection. In addition, there are challenges in utilizing other tasks to enhance prediction accuracy. The resolution of these issues is crucial for constructing models of depression detection.

**Methods:**

In this paper, we propose a multi-task representation learning based on vision and audio for depression detection model (DepITCM).The model comprises three main modules: a data preprocessing module, the Inception-Temporal-Channel Principal Component Analysis Module(ITCM Encoder), and a multi-task learning module. To efficiently extract rich feature representations from audio and video data, the ITCM Encoder employs a staged feature extraction strategy, transitioning from global to local features. This approach enables the capture of global features while emphasizing the fusion of temporal, channel, and spatial information in finer detail. Furthermore, inspired by multi-task learning strategies, this paper enhances the primary task of depression classification by incorporating a secondary task (regression task) to improve overall performance.

**Results:**

We conducted experiments on the AVEC2017 and AVEC2019 datasets. The results show that, in the classification task, our method achieved an F1 score of 0.823 and a classification accuracy of 0.823 on the AVEC2017 dataset, and an F1 score of 0.816 and a classification accuracy of 0.810 on the AVEC2019 dataset. In the regression task, the RMSE was 6.10 (AVEC2017) and 4.89 (AVEC2019), respectively. These results demonstrate that our method outperforms most existing methods in both classification and regression tasks. Furthermore, we demonstrate that the model proposed in this paper can effectively improve the performance of depression detection when using multi-task learning.

**Discussion:**

Although depression detection through multimodality has shown good results in previous studies. However, multi-task learning can utilize the complementary information between different tasks. Therefore, our work combines multimodal and multi-task learning to improve the accuracy of depression detection. Previous studies have mostly focused on the extraction of global features while ignoring the importance of local features. Based on the problems of previous studies, we have made corresponding improvements to provide a more comprehensive and effective solution for depression detection.

## 1 Introduction

Depression is a common mental illness. According to the World Health Organization (WHO), depression will be the leading problem in the global burden of disease by 2030 (1). Depression tends to exhibit symptoms such as mental ill-health, depressed mood, diminished interest and pleasure, and in severe cases, patients with depression may exhibit symptoms of self-harm and suicide (2). Therefore, early detection and treatment of depressed individuals is crucial. To assist clinicians effectively, the initial step in treating depression involves assessing whether an individual is depressed. Currently, the clinical diagnosis of depression relies on non-laboratory methods, which are conducted through structured or semi-structured interviews, during which the clinician assesses the patient’s psychological state based on standardized depression scales (e.g., the HAMD) (3) and communicates with the patient. However, this method is subjective and influenced by the clinician’s experience and the patient’s cooperation. In addition to linguistic differences, depressed individuals exhibit nonverbal characteristics such as painful expressions, low voice tone, drooping corners of the mouth, and slow movements. These subtle changes, described using the Facial Action Coding System (FACS), can be challenging for clinicians to detect accurately. The advent of deep learning techniques offers promise in overcoming these challenges.

This paper explores the application of deep learning techniques to integrate visual and audio features in depression detection. Currently, depression detection is mainly categorized into unimodal and multi-modal based depression recognition studies.

In single-modality-based depression recognition research, Melo et al. (4) introduced the Multiscale Spatiotemporal Network(MSN), An Affective Disorder Estimation (ADE) method for depression detection using 3D convolutional neural networks (CNNs).MSN initially applies multiscale convolution to encode spatio-temporal information and then integrates the multiscale information. Carneiro et al. (5) introduced the Maximization and Differentiation Network(MDN), a method that captures smooth facial changes using maximization blocks and encodes sudden facial change difference blocks to explore multiscale information. Jiang et al. (6) introduced an automatic classification method for depressed speech and analyzed the classification performance of speech features such as rhyme and spectrum in depression recognition. Dong et al. (7) introduced a hierarchical depression detection model. The model extracts features through a pre-trained deep residual network (ResNet). Although the above method focuses on global features, it lacks the extraction of local features. Tao et al. (8) introduced an efficient, low-covariance multimodal integrated spatio-temporal converter framework(DepMSTAT), a method for depression detection using spatio-temporal attention. DepMSTAT, although it takes into account spatio-temporal information in the data, ignores the fact that the different channels represent different objects and should be assigned different weights. In the related work summarized above, we found that previous studies have mainly focused on the extraction of global features and neglected the importance of local features, and fewer studies have given equal importance to both.

In multi-modality-based depression recognition research, Ceccarelli et al. (9) introduced the Adaptive Nonlinear Judgment Classifier, a decision-level fusion strategy based on feed-forward neural networks. Yin et al. (10) proposed a multimodal approach for depression detection using a recursive neural structure that integrates visual, audio, and textual modalities, validated with the AVEC2019 dataset. Fang et al. (11) introduced a multimodal fusion model with multi-level attention mechanism (MFM-Att), a method that combines Long Short-Term Memory Forgetting Networks (LSTMs) and Feature Fusion Networks (FFNs) for depression detection. Wang et al. (12) introduced multi-modal feature layer fusion model based on attention mechanisms (MFF-Att), a tandem model that uses multilayer CNNs and LSTMs. In the related work summarized above, multimodal tasks have shown better recognition results in depression recognition. However, these studies neglected the existence of certain complementary information between different tasks, which can improve the accuracy and generalization ability of the model.

This paper explores the application of deep learning techniques to integrate visual and audio features in depression detection. Previous studies have mainly focused on the extraction of global features, ignoring the importance of local features, and few studies have paid equal attention to the two. At the same time.in the process of local feature extraction, few studies focus on features in the three dimensions of time channel and space at the same time. The channel features provide fine-grained information of visual and audio data, and the temporal features of facial expressions express the trend of emotional changes. Spatial features describe the interrelationship between these details in different parts to convey the emotional state of a given moment, Therefore, it is an urgent problem to be solved in the detection of depression while emphasizing the global features and enhancing the extraction and integration of local features. The main contributions of this paper are as follows:

- First, we propose a multi-task representation learning based on vision and audio for depression detection model (DepITCM).The model features the Inception-Temporal-Channel Principal Component Analysis Module(ITCM Encoder) as its pivotal component, which integrates the Inception Dilated Convolution and the Temporal-Channel Principal Component Analysis Module (TCPCA). The TCPCA module comprises Temporal Attention and Channel Principal Component Analysis (CPCA). The proposed method extracts global features while emphasizing the key semantic information of visual and audio data, focusing on the fusion of temporal, channel, and spatial dimensions. This approach enables a better understanding of individuals’ emotional states and facilitates early detection and intervention in depressive symptoms.
- Secondly, this paper also adopts a multi-task representation learning strategy, because a current study (13) has demonstrated that multi-task learning can improve the effectiveness of depression detection. Therefore, we apply both multimodal and multi-task learning to the DepITCM model, leveraging the complementary information from different tasks to enhance the accuracy of depression detection.
- Finally, we validate on the AVEC2017 and AVEC2019 datasets. We demonstrate the performance of the proposed method in multi-task learning. In addition, we conducted ablation experiments to verify the effectiveness of each component of the model proposed in this paper.

**Figure 1:**
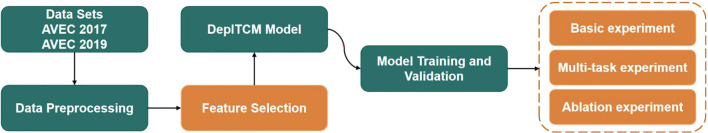
The research structure of this paper.

The research structure of this paper is shown in **Figure 1**. The rest of the sections are described below. Section 2 describes the dataset and the proposed depression detection method. The corresponding experimental results are given in Section 3. The proposed method is discussed in Section 4. Finally, Section 5 summarizes the whole paper.

## 2 Materials and methods

### 2.1 Datasets and preprocessing

Although previous studies (14, 15), have been developed for the AVEC2017 and AVEC2019 datasets, none of them explored the depression detection task from a non-verbal perspective, as most of the approaches used textual recordings in their models. In this paper, we focus on depression detection using visuals and sounds.

#### 2.1.1 AVEC 2017 dataset

The AVEC2017 (DAIC-WOZ) dataset (16) is a multimodal dataset consisting of clinical interviews for depression assessment. The dataset consists of 189 subjects, where the training, validation, and test sets contain data from 107, 35, and 47 participants, respectively. The visual data for each subject included 2D and 3D facial keypoints (Facial Landmarks), coordinates of eye gaze (Gaze), histogram of facial orientation gradient features (HOG), head pose data (Head Pose, HP), and facial action units (Action Units, AU); the audio data were extracted using the COVAREP toolbox (17) extracted features of the original audio file, recorded at 16 kHz, including both COVAREP and FORMANT features.

We refer to (11) for preprocessing the AVEC2017 dataset. For the audio preprocessing process, firstly for each subject, COVAREP features were extracted from the raw data and processed based on the conversation start and end times as well as the participant’s conversation tags. Then, the COVAREP features were normalized to ensure a similar scale and range between different features. Finally, non-speech frames and lines with response durations of less than 1 second were removed. To ensure the consistency of the data, it was padded with zeros to give it a uniform duration. For the visual preprocessing process, the data is first subsampled at 0.3-second intervals. Then, frames that were not successfully detected were deleted and the number of frames in each subject’s data was counted, recording the maximum and minimum frames to ensure consistency when processing and analyzing the data.

#### 2.1.2 AVEC 2019 dataset

The AVEC2019 (E-DAIC) dataset (18) is an extended version of AVEC2017 (DAIC-WOZ). The dataset consists of 275 subjects with 163, 56, and 56 samples in the training, validation, and test sets, respectively. The AVEC2019 dataset includes head pose data (Head Pose, HP), facial action units (Action Units, AU), and coordinates of eye gaze (Gaze) among the visual features; the audio data use dataset provided by the extended Geneva Minimalistic Acoustic Parameter Set (eGeMAPS) (19), which contains 88 feature values of the audio, covering acoustic dimensions such as spectrogram, power, cepstrum and speech quality of the audio. In addition to this, the Mel-scale Frequency Cepstral Coefficients (MFCC) of the audio are also used. For the AVEC2019 preprocessing approach, this paper uses the method provided by the AVEC 2019 DDS (20). For audio modalities we used Extended Geneva Minimalistic Acoustic Parameter Set(eGeMAPS) and MFCC; for visual modalities we used head pose data (Head Pose, HP), facial action units (Action Units, AU), and coordinates of eye gaze (Gaze).

The labeling files for both the AVEC2017 and AVEC2019 datasets contain PHQ scores for all samples, with scores ranging from 0 to 24. This also includes a binary value for whether or not the sample is depressed, with a PHQ value in the range of [10, 24] representing having a depressive disorder, and a score in the range of [0, 9] being normal.

### 2.2 Feature selection

After completing the preprocessing, the features in such samples have very little variation and contribute very little to the classification and regression tasks, considering that there are cases such as noise or long pauses in the data, and they do not have enough variation across samples to distinguish between different values of the target variable. Therefore, in this study, we measure the importance of the features by calculating the variance of the features and filtering out the features with low variance. The formula for calculating the variance of features is:


(1) $$\text{Var}(X)=\frac{1}{n}\sum_{i=1}^{n}{\left(X_{i}-\mu \right)}^{2}$$

Where Var(*X*) denotes the variance of feature *X*, *X_i_* is the feature value of the *i*th sample, *µ* is the mean value of feature *X*, and *n* is the total number of samples. In this paper, the threshold is set to 10 to filter out features with variance lower than 10, retaining only high variance features that contribute more significantly to the classification and regression tasks.

### 2.3 Proposed methodology

**Figure 2:**
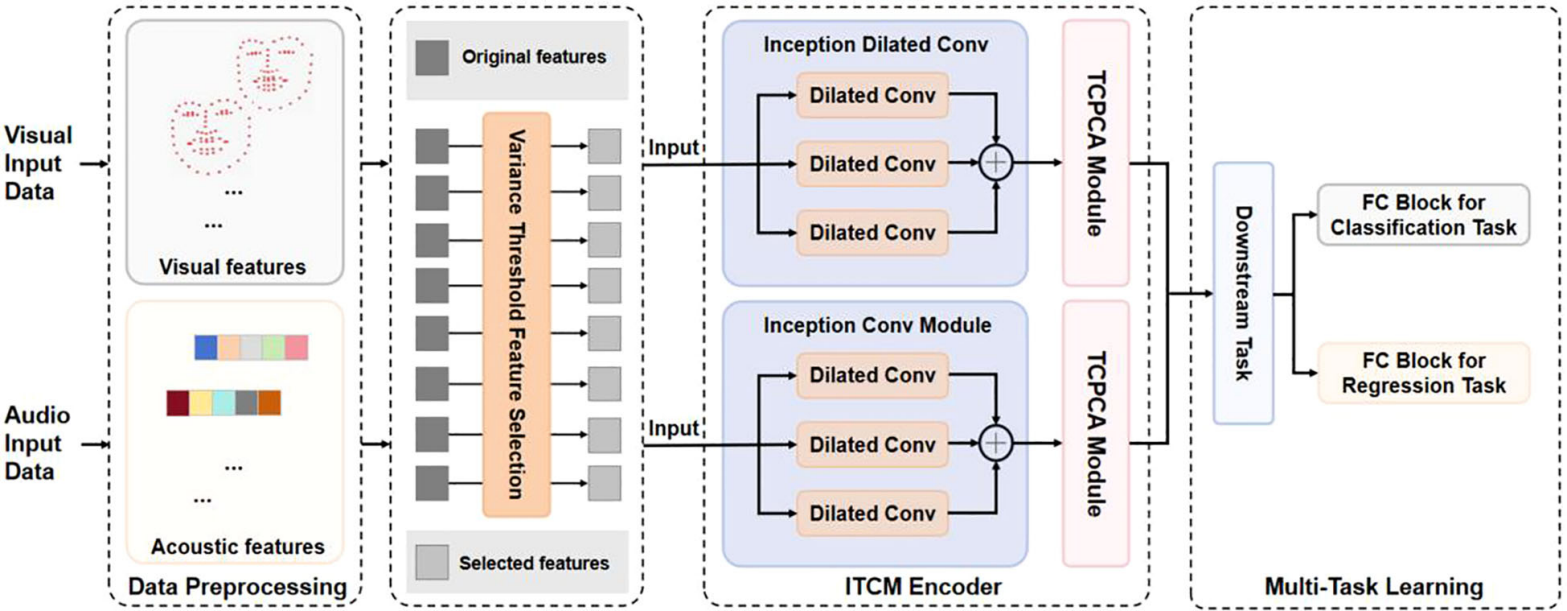
The general framework of DepITCM. The raw data first undergoes a data preprocessing stage, then global and local key features are extracted using ITCM Encoder. Finally the multi-task learning module receives the feature sequence representation and performs regression and classification tasks.

Our proposed research framework is shown in **Figure 2**. After completing data preprocessing and feature screening, we constructed DepITCM to accomplish the task of automatic identification of depression. This section describes the architectural approach of DepITCM. DepITCM consists of an ITCM encoder and a multi-task learning module. Among them, the role of the ITCM encoder is to extract the visual and audio features. To leverage the complementarity between the tasks and improve the overall performance of the model, we also added the multi-task learning module to DepITCM.

#### 2.3.1 ITCM Encoder

**Figure 3:**
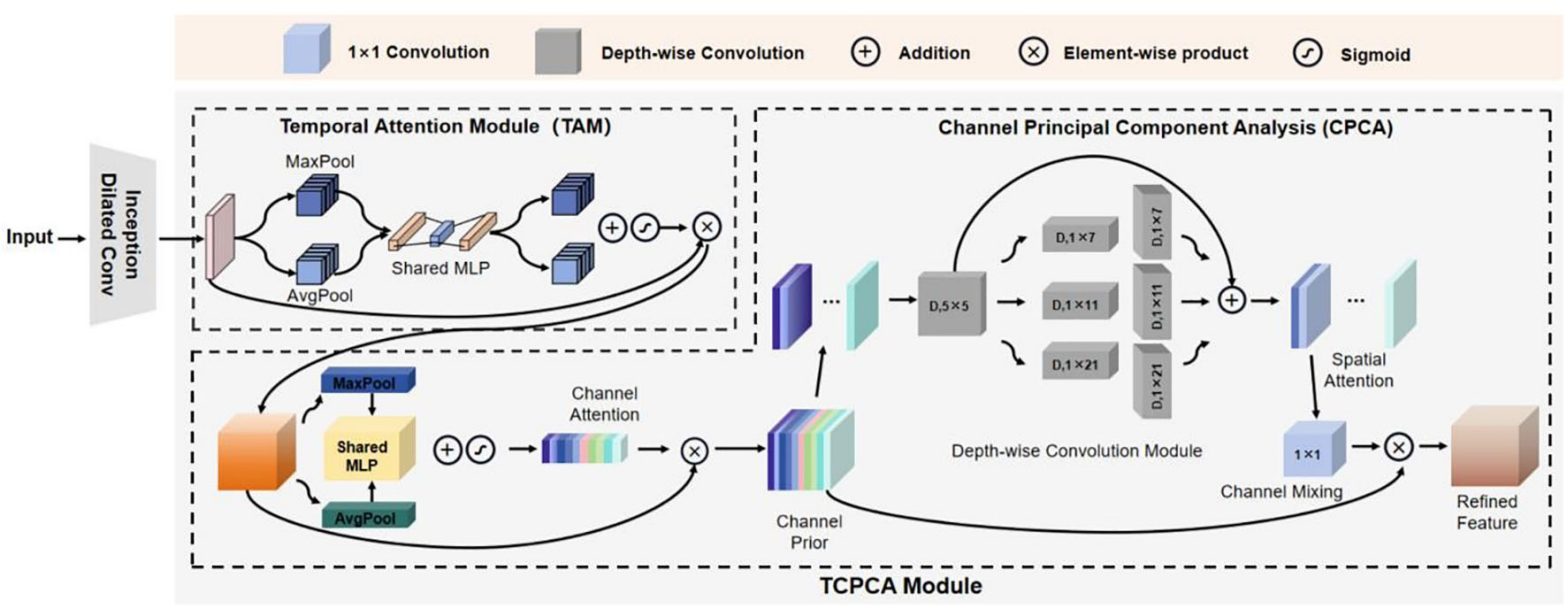
The detailed structure of ITCM encoder.

The structure of the feature extraction module ITCM Encoder is shown in **Figure 3**. The ITCM Encoder consists of two main modules: the Inception Dilated Conv and the TCPCA module. The Inception Dilated Conv is designed to extract global features of the data, synthesizing multi-scale long-range information from visual and audio modalities. The TCPCA module is then used to further extract key information, focusing particularly on the fusion of information in the temporal, channel, and spatial dimensions. This approach enables our model to effectively mine feature information from both audio and visual data, providing a more comprehensive understanding and characterization of the emotional features of depressed patients.

##### 2.3.1.1 Inception Dilated Convolution

To learn the global information, we propose Inception Dilated Convolution, which introduces expansion factors to the convolutional layer (Conv) in the Inception structure (21) to complement the global information and increase the model diversity. The information is aggregated by the Maxpool layer after the Inception Dilated Conv. Taking visual modality as an example, Inception Dilated Convolution uses parallel 1 × 1 convolution, 3×3 convolution, and 5×5 convolution, and 3×3 Maxpool layer for computation as follows:


(2) $$\begin{array}{c} IDC\left(x\right)=\text{Contact }(f^{1\times 1}\left(x\right),f^{3\times 3}\left(x\right),f^{5\times 5}\left(x\right),\\ f^{1\times 1}\left(\text{Maxpool }(x\right))) \end{array}$$

where x denotes the input of Inception Dilated Convolution, Contact refers to the splicing of features by dimension, and $f^{1\times 1}$ , $f^{3\times 3}$ , and $f^{5\times 5}$ denote 1×1 convolution, 3×3 convolution, and 5×5 convolution, respectively.

The final feature Feature is calculated by combining stacking of Inception Dilated Convolution and Maxpool as follows:


(3) Feature(x)=maxpool (IDR(x))

##### 2.3.1.2 TCPCA module

To enhance local information, various local attention mechanisms have been proposed. Squeeze-and-Excitation Networks (SENet) (22) explicitly establishes dependencies between feature channels to facilitate learning relationships between different feature channels. Convolutional Block Attention Module (CBAM) (23), while integrating both channel attention and spatial attention, enforces a consistent distribution of spatial attention across all channels of an output feature. In contrast, Channel Prior Convolutional Attention (CPCA) (24) employs multi-scale deeply separable convolutional modules to constitute spatial attention, which can dynamically distribute the attention weights in both channel and spatial dimensions, thus better integrating the information of feature channels and spatial locations. In this regard, we adopt CPCA to enhance local features and solve the problem of information loss caused by simple crosstalk, so that the model can better focus on local features. These attention mechanisms construct a mapping method between global information and locally important information, by which local features are extracted and used to enhance the original features. Meanwhile, these attention mechanisms can be directly integrated into the backbone network without the need for a separate network.

Considering the importance of temporal information in sequence learning tasks, we introduced the temporal attention module before CPCA. This module captures the dependencies in the temporal dimension, thereby enhancing temporal information extraction and improving detection accuracy. Additionally, we conducted ablation experiments on CBAM and CPCA modules to verify their contributions. The TCPCA module is specifically calculated as follows:

Given a feature sequence $F_{in}$ , the TCPCA module sequentially calculates the temporal attention module $\text{TAM}\left(F_{in}\right)$ , channel attention module $\text{CAM}\left(F_{in}\right)$ , and spatial attention module $\text{SAM}\left(F_{in}\right)$ . The entire computation process is as follows:


(4) $$F^{\prime }=\text{TAM}\left(F_{in}\right)⊗F$$

(5) $$F^{\prime \prime }=\text{CAM}\left(F_{in}\right)⊗F^{\prime }$$

(6) $$F^{\prime \prime \prime }=\text{SAM}\left(F_{in}\right)⊗F^{\prime \prime }$$

Here, $F,F^{\prime },F^{\prime \prime }$ respectively denote the input features after processing with the temporal attention module (TAM), channel attention module (CAM), and spatial attention module (SAM). The operator ⊗ represents element-wise multiplication.

The temporal attention module (TAM) is calculated as follows:


(7) $$\begin{array}{c} \text{TAM}\left(F_{in}\right)=\sigma \;(\text{MLP}\left({\text{AvgPool}}_{\text{time}}\left(F_{in}\right)\right)\\ +\text{MLP}\left({\text{MaxPool}}_{\text{time}}\left(F_{in}\right)\right) \end{array})$$

Where $F_{in}$ denotes the input to the TAM, *σ* denotes the sigmoid activation function, *MLP* denotes the fully connected neural network, and ${\text{AvgPool}}_{\text{time}}$ and ${\text{MaxPool}}_{\text{time}}$ denote average pooling and maximum pooling operations along the temporal dimension, respectively. The CPCA attention mechanism is mainly composed of two key components, namely, the Channel Attention Module (CAM) and the Spatial Attention Module (SAM). The processing flow of the CPCA module is as follows:


(8) $$\begin{array}{c} \text{CAM}\left(F_{in}\right)=\sigma (\text{MLP}\left({\text{AvgPool}}_{\text{channel}}\left(F_{in}\right)\right)\\ +\text{MLP}\left({\text{MaxPool}}_{\text{channel}}\left(F_{in}\right)\right) \end{array})$$

Where $F_{in}$ denotes the input to CPCA, *σ* denotes the sigmoid activation function, MLP denotes the fully connected neural network, and ${\text{AvgPool}}_{\text{channel}}$ and ${\text{MaxPool}}_{\text{channel}}$ denote the average pooling and maximum pooling along the channel dimension. The SAM is computed as follows:


(9) $$\text{SAM}(F_{\text{in}})=\text{Con}{\text{v}}_{1\times 1}\left(\sum_{i=0}^{3}\text{Branc}{\text{h}}_{i}(\text{DwConv}(F_{\text{in}}))\right)$$

where *DwConv* represents a deep convolution, and ${\text{Branch}}_{i}$ , i∈{0,1,2,3} represents the *i*th branch.

### 2.4 Multi-task learning

Considering that the shared feature extraction layer can improve the richness of feature representation and the complementary information between different tasks can improve the generalization ability of the model, in this regard, we added an auxiliary task (regression task) to augment the main task of depression classification. For the purpose of multi-task learning, after feature extraction, the obtained features are sent to the FC block to perform the multi-task learning task.

The multi-task loss function in the training phase can be expressed as:


(10) $$\text{Loss}=a\cdot L_{\text{re}}+b\cdot L_{\text{cl}}$$

where $L_{\text{re}}$ and $L_{\text{cl}}$ are the loss functions for PHQ-8 regression and binary classification, respectively. a and b are designed to utilize the coefficients between these two tasks, which can be set as hyperparameters. In this study, a is set to 0.7 and b is set to 0.3. Specifically, we use the commonly used cross-entropy loss function as the loss function for the binary classification task, which can be expressed as:


(11) $$L_{\text{c}1}=\frac{1}{N}\sum_{i=1}^{N}\left[y_{i}\text{log }({\hat{y}}_{i})+(1-y_{i})\text{ log}(1-{\hat{y}}_{i})\right]$$

where *N* is the number of samples, $y_{i}$ is the true label of the i -th sample, and ${\hat{y}}_{i}$ is the predicted probability of the *i*-th sample. The loss function of the PHQ-8 regression can be expressed as:


(12) $$L_{\text{re}}=\frac{1}{N}\sum_{i=1}^{N}{\left(y_{i}-{\hat{y}}_{i}\right)}^{2}$$

where *N* is the sample size, $y_{i}$ and ${\hat{y}}_{i}$ the true and predicted PHQ-8 scores of the *i*-th sample, respectively.

## 3 Results

To validate the effectiveness of DepITCM, we conducted experimental validation on the AVEC2017 and AVEC2019 datasets. Additionally, we aimed to demonstrate that the proposed model is applicable to depression detection and that the multi-task learning strategy can improve the accuracy of depression detection. We conducted comparison experiments, multi-task learning experiments, and ablation experiments. The model proposed in this paper is built using the Keras framework. All experiments are trained and tested on NVIDIA GeForce A800 GPUs. For the classification task, we use accuracy, F1 score, recall, and precision as evaluation criteria; for the regression task, MAE and RMSE are used as evaluation criteria.

### 3.1 Comparison with existing models

For the AVEC 2017 dataset, most of the previous studies (12) only used Action Units and 3D facial landmark for depression detection, unlike these methods, we also captured two visual features, Head Pose and Eye Gaze, to enrich the feature representation; for audio modality this paper captured COVAREP and FORMANT two features; for the AVEC 2019 dataset, we follow the methods provided by AVEC 2019 DDS to capture visual and audio features. **Tables 1**, **2** show the results of performance comparison between the method proposed in this paper and other methods on AVEC 2017 and AVEC 2019 datasets, respectively.

**Table 1:** Comparison of the performance of DepITCM on AVEC 2017 datasets with other methods in recent years.

Method	Classification				Regression	
	F1	Precision	Recall	Accuracy	RMSE	MAE
Ringeval et al. (25)	–	–	–	–	6.62	5.52
Pan et al. (26)	–	–	–	–	5.49	4.55
Fang et al. (11)	–	–	–	–	**5.20**	**4.12**
Kumar et al. (27)	0.63	–	–	–	6.35	5.38
Gimeno et al. (28)	0.67	0.68	0.66	–	–	–
Wei et al. (29)	0.61	0.78	0.50	–	6.06	5.06
Tiwary et al. (30)	0.746	0.667	0.667	0.771	–	–
**DepITCM (Ours)**	**0.823**	**0.860**	**0.801**	**0.823**	6.10	5.21

Bold data indicates the best performing results.

**Table 2:** Comparison of the performance of DepITCM on AVEC 2019 datasets with other methods in recent years.

Method	Classification				Regression	
	F1	Precision	Recall	Accuracy	RMSE	MAE
Ringeval et al. (18)	–	–	–	–	6.37	–
Saggu et al. (14)	–	–	–	–	5.36	**4.32**
Sun et al. (31)	–	–	–	–	**3.78**	–
Gimeno et al. (28)	0.56	0.59	0.58	–	–	–
Li et al. (32)	–	–	–	0.79	4.80	4.58
**DepITCM (Ours)**	**0.816**	**0.813**	**0.806**	**0.810**	4.89	4.62

Bold data indicates the best performing results.

### 3.2 Multi-task learning experiments

To verify whether the proposed model benefits from multi-task learning, we compared the results of single-task learning and multi-task learning. In the regression task, we classified the regression output according to the five levels of the official PHQ-8 scale (0-4, 5-9, 10-14, 15-19, 20 and above), and these levels served as the evaluation criterion for the regression task. As shown in **Table 3**, multi-task representation learning outperforms single-task learning in both classification and regression tasks. This indicates that shared features have a positive impact on both classification and regression tasks, demonstrating that the proposed model benefits from multi-task learning and effectively enhances depression detection performance.

**Table 3:** Comparison of Single-Task and Multi-Task results based on accuracy, RMSE, and other metrics.

Tasks	AVEC 2017				AVEC 2019			
	F1	Acc	RMSE	MAE	F1	Acc	RMSE	MAE
**Single-Task**	0.815	0.811	6.18	5.28	0.796	0.803	4.96	4.71
**Multi-Task**	**0.823**	**0.823**	**6.10**	**5.21**	**0.816**	**0.810**	**4.89**	**4.62**

Where Single-Task refers to training the PHQ-8 regression task or classification task alone, and Multi-Task refers to training both the PHQ-8 regression task and classification task. Acc refers to the accuracy rate. Bolded data indicate the best performing results.

### 3.3 Ablation experiments

#### 3.3.1 Verification of the effectiveness of each module

To validate the effect of the DepITCM model on depression recognition ability, we designed four model structures to explore the importance of each module in DepITCM. (i) Inception: We use the Inception structure to extract modality-specific features, then concatenate all features and finally apply a fully connected layer for depression detection. This model is primarily used to verify the performance of the standard Inception module in feature extraction; (ii) Inception+TCPCA: This model extracts features using Inception, then fuses the extracted features through the TCPCA module for depression detection. It is used to validate the role of the TCPCA module in enhancing local features; (iii) Inception Dilated Convolution (IDC): This model extracts features using the IDC module, concatenates the extracted features, and then applies a fully connected layer for depression detection. It is used to verify the effectiveness of the IDC module in capturing global features; (v) DepITCM, which consists of two main modules, IDC and TCPCA. In addition to this, we also verified the effect of replacing the CPCA module in DepITCM with CBAM.

**Table 4** presents the depression detection performance of four different model architectures on the AVEC2017 and AVEC2019 datasets. The performance of Inception for depression detection is relatively poor, likely due to the sparsity of convolutions and the limitations of the receptive field imposed by the convolution kernel size, making standard convolutions unable to effectively capture global dependencies in time-series data. Next, by comparing pairs of models, namely Inception vs. Inception+TCPCA and Inception vs. IDC, significant performance improvements can be observed in **Table 4**. The IDC module increases the receptive field through dilated convolutions, effectively capturing global temporal patterns without increasing computational complexity. The TCPCA module enhances local feature representations by combining temporal attention and channel feature enhancement, making the features more representative. This demonstrates that the IDC and TCPCA modules complement each other in the DepITCM model and are key to its performance improvement.

**Table 4:** Performance of different structural models on AVEC 2017 and AVEC 2019 datasets.

Datasets	Method	Classification				Regression	
		F1	Precision	Recall	Accuracy	RMSE	MAE
**AVEC 2017**	**Inception**	0.705	0.702	0.716	0.693	6.97	6.09
	**Inception+TCPCA**	0.774	0.783	0.781	0.784	6.31	5.44
	**IDC**	0.736	0.746	0.725	0.721	6.74	5.83
	**DepITCM-CBAM (Ours)**	0.798	0.805	0.783	0.786	6.30	5.36
	**DepITCM (Ours)**	**0.823**	**0.860**	**0.801**	**0.823**	**6.10**	**5.21**
**AVEC 2019**	**Inception**	0.686	0.692	0.701	0.668	6.02	5.39
	**Inception+TCPCA**	0.776	0.782	0.762	0.764	5.23	4.92
	**IDC**	0.728	0.733	0.732	0.711	5.61	5.18
	**DepITCM-CBAM (Ours)**	0.798	0.805	0.783	0.786	6.30	5.36
	**DepITCM (Ours)**	**0.816**	**0.813**	**0.806**	**0.810**	**4.89**	**4.62**

Bold data indicates the best performing results.

#### 3.3.2 The impact of TAM and CPCA order on performance

To further investigate the impact of the sequence of the TAM and CPCA modules on model performance, we designed two experiments: one where TAM is placed before CPCA (TAM-CPCA), and another where CPCA is placed before TAM (CPCA-TAM). The experimental results are presented in **Table 5**. The results demonstrate that the model achieves better performance when TAM precedes CPCA, indicating that enhancing temporal features first with TAM facilitates more efficient optimization of channel and spatial features by the subsequent CPCA module.

**Table 5:** The impact of TAM and CPCA order on performance.

Datasets	Order	Classification				Regression	
		F1	Precision	Recall	Accuracy	RMSE	MAE
**AVEC 2017**	**CPCA-TAM**	0.802	0.845	0.780	0.795	6.30	5.50
	**TAM-CPCA**	**0.823**	**0.860**	**0.801**	**0.823**	**6.10**	**5.21**
**AVEC 2019**	**CPCA-TAM**	0.781	0.803	0.775	0.790	5.24	4.89
	**TAM-CPCA**	**0.816**	**0.813**	**0.806**	**0.810**	**4.89**	**4.62**

Bold data indicates the best performing results.

## 4 Discussion

Previously conducted studies have illustrated the feasibility of using vision and audio for depression detection (5–8), have focused on the extraction of global information, which is consistent with our study. However, they neglected the importance of local features. The method proposed by (9) has the ability to fuse the spatio-temporal information in the data, compared with the method proposed in this paper, we not only focus on the spatio-temporal information, but also emphasize the extraction of the channel information, and we can dynamically allocate the attention weight in the spatial dimension, to refine the extraction of the local features. Although (12) focuses on both global and local information, it mainly concentrates on the temporal dimension and has relatively few local features extracted. To address these issues, we propose DepITCM. DepITCM can comprehensively integrate global information and extract features in time, channel, and spatial dimensions. In addition, we design ablation experiments to demonstrate the effectiveness of each module in DepITCM.

In addition, we designed ablation experiments to demonstrate the effectiveness of each module in DepITCM. In the ablation experiments, we set up control groups for the main modules, IDC and TCPCA. The results show that IDC and TCPCA, as feature encoders, effectively extract global and local features of the modalities, significantly improving the performance of the DepITCM model.

We also demonstrated the superiority of multi-task learning over single-task learning. By incorporating the multi-task learning strategy into the model, we can more effectively utilize the complementary information between different tasks, further enhancing the accuracy and generalization ability of depression detection. Experimental results indicate that DepITCM with multi-task learning outperforms single-task learning across different tasks, proving the effectiveness of multi-task learning and its role in improving model performance.

In practical applications, DepITCM has the potential to be integrated into systems related to depression treatment monitoring, supplemental assessments, and online health assessments. Its multimodal nature allows the model to synthesize and analyze multiple signals in real time in a clinical setting, thereby supporting physicians in more accurate diagnosis. For example, in a clinical setting, the model can be used to assist in the early screening of patients with depression and provide objective quantitative metrics. In addition, the multi-task learning strategy enhances the model’s adaptability and generalization ability in different environments, enabling it to meet the needs of different clinical scenarios and patients. This is of great significance to the field of mental health, especially the objectivization and real-time nature of depression detection.

Compared to current state-of-the-art methods, DepITCM outperforms these methods in classification metrics on the AVEC 2017 and AVEC 2019 datasets, but does not fully surpass all advanced methods in regression task evaluation metrics. For example, Fang et al. (11) proposed MFM-Att, which strengthens the interaction and complementarity between modalities through multi-level fusion (FFN + AttFN). This strategy extracts information from different levels, fully utilizing the complementary nature of different modalities and providing richer emotional information. In contrast, our method focuses more on feature extraction, employing a staged feature extraction strategy that first extracts global features and then local features, allowing for a more comprehensive understanding and representation of the emotional features of depression patients. As shown in **Table 4**, the staged feature extraction strategy achieved good results, demonstrating its effectiveness. Pan et al. (26) proposed the Audio-Visual Attention Network (AVA-DepressNet), which, due to differences in pretraining objectives and datasets, uses a multi-stage pretraining strategy. In the first stage, the visual encoder is pretrained using the CelebA dataset to capture general facial features, and in the second stage, the model is further fine-tuned using the AVEC dataset to capture depression-related features. Moreover, the multi-stage pretraining objectives effectively enhance the model’s ability to represent both general features and task-specific features. As a result, AVA-DepressNet outperforms our method in the regression task. Furthermore, Saggu et al. (14) proposed DepressNet, which introduces text modality due to the modality differences, providing more comprehensive information support that allows it to capture richer depressive features. As a result, DepressNet outperforms our method in the regression task. The text modality can compensate for the limitations of visual and audio modalities in terms of overt behavioral features by capturing implicit emotional tendencies, psychological hints, and other hidden information in language expression.

Currently, DepITCM only integrates visual and audio modalities. In the future, it may be beneficial to incorporate additional modalities, such as text. Previous studies (10, 33–37), have shown that combining visual, audio, and text modalities can provide multi-angle deep learning models for depression detection. Therefore, how to effectively integrate this multimodal information remains an important issue worth further exploration. Moreover, although the model’s performance has shown significant improvements in the experiments, further validation on broader datasets and in real-world scenarios is needed to ensure its reliability.

## 5 Conclusion

To develop a method that can help clinicians detect depression objectively and quickly, this paper constructs a depression detection model (DepITCM) based on multi-task representation learning with vision and audio. Among them, the IDC integrates the global information of the data, while the TCPCA module effectively enhances the local information, extracts the temporal information, and integrates the channel and spatial features, which enhances the representational ability of DepITCM. The experimental results show that the DepITCM model achieves a significant performance improvement compared with the existing methods in recent years, and can comprehensively extract multi-modal information. In addition, in the ablation experiments, we verified that the two sub-modules have a positive effect on the generalization ability of DepITCM, proving the effectiveness of the proposed sub-modules. Finally, DepITCM outperforms single-task learning in all multi-task learning experiments, proving that the shared feature extraction layer can improve the richness of feature representation. In conclusion, the method proposed in this paper advances the development of intelligent systems for mental health to a certain extent, and the method is promising as a potential tool for clinicians and researchers. However, the method proposed in this paper still has room for improvement in recognition accuracy. Future work will focus on optimizing the model structure and incorporating additional modal information.

## Data Availability

Publicly available datasets were analyzed in this study. This data can be found here: AVEC 2017: Official website: https://dcapswoz.ict.usc.edu/; Download method:; Download Printing Protocol: https://dcapswoz.ict.usc.edu/wwwutil_files/DAICWOZDEP_EULA.pdf; Sign and send to boberg@ict.usc.edu. AVEC 2019: Official website: https://www.ihp-lab.org/resources/; Download method:; Download Printing Protocol: https://www.ihp-lab.org/downloads/Extended-DAIC-BLANK_EULA.pdf; Sign and send to boberg@ict.usc.edu.
